# Cholera Outbreak, Laos, 2007

**DOI:** 10.3201/eid1604.091493

**Published:** 2010-04

**Authors:** Noikaseumsy Sithivong, Hidemasa Izumiya, Khampheuy Munnalath, Traykhouane Phouthavane, Khampheng Chomlasak, Lay Sisavath, Arounnapha Vongdouangchanh, Phengta Vongprachanh, Haruo Watanabe, Makoto Ohinishi

**Affiliations:** National Center for Laboratory and Epidemiology, Vientiane, Laos (N. Sithivong, K. Munnalath, T. Phouthavane, K. Chomlasak, L. Sisavath, A. Vongdouangchanh, P. Vongprachanh); National Institute of Infectious Diseases, Tokyo, Japan (H. Izumiya, H. Watanabe, M. Ohinishi)

**Keywords:** bacteria, cholera, *Vibrio cholerae*, Laos, cholera, bacteria, letter

**To the Editor:** Cholera is a major public health problem in countries where access to safe water and adequate sanitation cannot be guaranteed for all. *Vibrio cholerae* serogroups O1 and O139 are the causative agents of cholera (*1*). One of the most powerful virulence factors in this organism is cholera toxin encoded by the *ctxAB* gene, located on the CTX prophage. *V. cholerae* O1 is classified into 2 biotypes, classical and El Tor. The El Tor type of *V. cholerae* O1 is responsible for the ongoing seventh worldwide pandemic of cholera (*2*). The sequence of *ctxB* of a certain strain has been believed to correspond to its biotype; that is, a biotype classical strain has classical type *ctxB,* and a biotype El Tor strain has El Tor type *ctxB*. However, recent research studies suggest that novel types of *V. cholerae* O1, hybrid strains, and altered El Tor or El Tor variant strains (*1*,*3*) are emerging. Altered El Tor or El Tor variant strains are biotype El Tor but produce classical cholera toxin (*3*,*4*). Recent reports suggest that this type of *V. cholerae* O1 is spreading to many areas of the world (*5*).

In December 2007–January 2008, a cholera outbreak occurred in Xekong Province in southeastern Laos, in the Mekong Basin. The first case of the outbreak was detected on December 23, 2007. The outbreak spread to 10 villages and lasted through January 2008. Specifically, in the Thateng District, 117 cases occurred and 2 deaths were reported. The sources of the outbreak were suspected to be regularly used water. In October 2007, 2 months before the outbreak, 3 sporadic cases of *V. cholerae* infection had been identified in Vientiane (the capital city) and Xaignabouri Province in north-central and northwestern Laos, respectively. The outbreak investigation in the Xekong Province identified no linkage between these sporadic cases and the outbreak cases.

In this study, we analyzed 18 *V. cholerae* isolates obtained in 2007: 3 were from patients with sporadic cases, and 15 were from the Xekong outbreak (13 from patients and 2 from water samples). All the isolates were serotype O1 Ogawa and biotype El Tor, but their *ctxB* types were classical, according to the method previously described (*6*). This finding indicates that they were the type of altered El Tor.

We used pulsed-field gel electrophoresis (PFGE) to investigate relationships between the isolates according to the PulseNet protocol (*7*). All 18 isolates from the sporadic cases and the outbreak in 2007 displayed profiles indistinguishable from each other (Figure). We also compared 2 additional *V. cholerae* O1 isolates, 1 from a patient in Vientiane in 1998 and another from a patient in Louangphabang in 2000 (Figure). The profiles of the isolates obtained in 1998 and 2000 clearly differed from those obtained in 2007. These results indicate that all isolates from sporadic and outbreak cases in 2007 were likely from the same source of contamination, although extensive epidemiologic investigation did not identify any common source.

**Figure:**
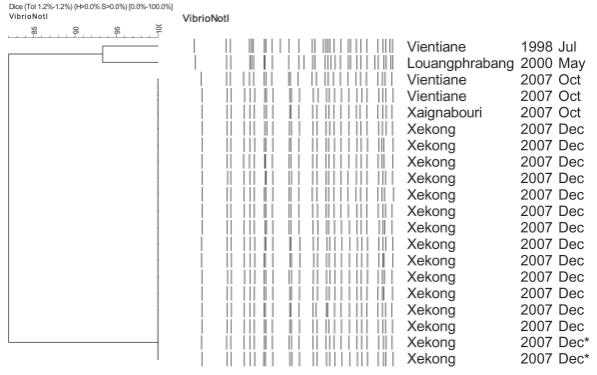
Dendrogram for *Not*I-digested pulsed-field gel electrophoresis profiles of *Vibrio cholerae* isolates, Laos, December 2007–January 2008. Origin of each isolate is shown on the right. *Water sample.

Nguyen et al. characterized the isolates from a cholera outbreak in Vietnam from late 2007 to early 2008 (*8*). Their report suggests that the isolates from the outbreaks in Vietnam and Laos shared the same elements of the CTX prophage. Our study suggests a common source for the strains of sporadic cases in Vientiane and Xaignabouri Province in October 2007 and those of the outbreak in Xekong Province in December 2007. Molecular typing suggests that a novel clone of *V. cholerae* O1 is being disseminated along the Mekong Basin. However, no epidemiologic association has been identified so far. Thus, a more extensive regionwide surveillance system is needed to identify and control *V. cholerae* infection in Laos and neighboring countries.
